# Goal-Directed Reasoning and Cooperation in Robots in Shared Workspaces: an Internal Simulation Based Neural Framework

**DOI:** 10.1007/s12559-018-9553-1

**Published:** 2018-04-14

**Authors:** Ajaz A. Bhat, Vishwanathan Mohan

**Affiliations:** 1 School of Psychology, University of East Anglia, Norwich, UK; 2 CSEE Department, University of Essex, Colchester, UK

**Keywords:** Spatial reasoning, Planning, Cooperation, Internal models, Body schema, Peripersonal space, Industrial assembly

## Abstract

From social dining in households to product assembly in manufacturing lines, goal-directed reasoning and cooperation with other agents in shared workspaces is a ubiquitous aspect of our day-to-day activities. Critical for such behaviours is the ability to spontaneously anticipate what is doable by oneself as well as the interacting partner based on the evolving environmental context and thereby exploit such information to engage in goal-oriented action sequences. In the setting of an industrial task where two robots are jointly assembling objects in a shared workspace, we describe a bioinspired neural architecture for goal-directed action planning based on coupled interactions between multiple internal models, primarily of the robot’s body and its peripersonal space. The internal models (of each robot’s body and peripersonal space) are learnt *jointly* through a process of sensorimotor exploration and then employed in a range of anticipations related to the feasibility and consequence of potential actions of two industrial robots in the context of a joint goal. The ensuing behaviours are demonstrated in a real-world industrial scenario where two robots are assembling industrial fuse-boxes from multiple constituent objects (fuses, fuse-stands) scattered randomly in their workspace. In a spatially unstructured and temporally evolving assembly scenario, the robots employ reward-based dynamics to plan and anticipate which objects to act on at what time instances so as to successfully complete as many assemblies as possible. The existing spatial setting fundamentally necessitates planning collision-free trajectories and avoiding potential collisions between the robots. Furthermore, an interesting scenario where the assembly goal is not realizable by either of the robots individually but only realizable if they meaningfully cooperate is used to demonstrate the interplay between perception, simulation of multiple internal models and the resulting complementary goal-directed actions of both robots. Finally, the proposed neural framework is benchmarked against a typically engineered solution to evaluate its performance in the assembly task. The framework provides a computational outlook to the emerging results from neurosciences related to the learning and use of body schema and peripersonal space for embodied simulation of action and prediction. While experiments reported here engage the architecture in a complex planning task specifically, the internal model based framework is domain-agnostic facilitating portability to several other tasks and platforms.

**Electronic supplementary material:**

The online version of this article (10.1007/s12559-018-9553-1) contains supplementary material, which is available to authorized users.

## Introduction

From dining together to intelligently passing the ball in a soccer game, humans reason about how to act together in shared spaces in a goal-oriented manner. Similarly, at an individual level, from a rat’s way-finding towards potential rewards in a complex maze [1] to a chimp’s use of a rake to reach an otherwise unreachable object [2], cognitive agents effortlessly plan actions in space and time to fulfil goals of the self (and the others). This fundamental ability to explore, identify, internalize and exploit possibilities afforded by the structure of one’s immediate environment is critical for any artificial agent to exercise purposeful and intelligent behaviour in a messy world of objects, choices, relationships and other acting agents. The inherent complexity of this problem is revealed when we face the challenge of enabling robots to operate in unstructured and shared environments. Concrete solutions in this context are sought in a broad range of domains from assisted living for elderly to the industrial assembly line, in general any environment where multiple agents need to operate in shared workspaces.

To this effect, the article presents an internal model based neural architecture for goal-directed reasoning and cooperation^1^ between multiple robots, validated in a real-world industrial assembly task: where robots operate jointly to realize goals like assembling a complex object from its constituent parts. The underlying challenge is to flexibly deal with the *spatially unstructured* and *temporally evolving* nature of the assembly scenario to enable the operating robots to reason continuously about *which objects to act on at what time instances* so as to *jointly or individually realize as many assemblies as possible*. Therefore, rather than considering the joint assembly as a mere collision avoidance problem between robots and objects, this article proposes a reasoning framework that dynamically plans robot movements to *maximize the assembly success rate* as well as avoids any obstacles during multi-robot operations. While the assembly setup serves as a compelling scenario to explore the problem and address the synergistic interaction between multiple subsystems involving *perception-action-learning-reasoning* to enable goal-oriented behaviours, the architecture itself is domain-agnostic facilitating portability to several other domains.

A variety of computational models for spatial representation and reasoning have been proposed in the robotics literature. For example, previous work [3–5] on task-sequence planning for cooperative robots, applied in tasks like inspection operations formulate planning as variations of the travelling salesman or task assignment problem. These works employ simulated annealing, harmony searches, consensus bundling and different non-linear optimization methods for near-optimal solutions [6]. In another recent study [7] on multi-robot cooperation involving pick and place tasks, authors propose combining part-dispatching rules to coordinate robots. This is realized by integrating a greedy randomized adaptive search procedure (GRASP) and a Monte Carlo strategy (MCS). Conventional approaches like rapidly exploring random tree (RRT) algorithms have also been applied to bin-picking tasks [8]. Toussaint and Georick [9] provide a planning method based on probabilistic inference and demonstrate on a dual arm humanoid in reaching tasks, albeit applicable in structured environments only. Most of the other models in literature are based on constraints, logical, algebraic or ontological approaches (see [10–13] for a summary). Apart from the fact that these approaches cannot manage numeric, topological and imprecise knowledge at the same time [10, 14–17], demonstrations of the capabilities of these approaches have still been restricted to tasks that are carried out in controlled static conditions. Further, the imposition of pre-programmed optimality constraints specific to bodily and environmental conditions renders the models inflexible and inapplicable while porting to new tasks: a desirable feature needing innovative solutions [18].

Previous works applying artificial neural networks in industrial assemblies has mainly focused on the tuning of process parameters (such as force control parameters) to deal with part variations and system uncertainties [19]. The major drawbacks to these methods, however, are that they are all highly specialized to specific problems and must be reworked whenever modifications are introduced to the assembly setup or the robot. Other machine learning techniques such as genetic algorithms have also been explored extensively for assembly sequence and scheduling optimization [20–26] but their application to actual robotic tasks is relatively limited with the exception to recent works by Marvel and colleagues [27, 28]. Their studies present genetic algorithm based self-optimization methods for parameter tuning which can predict when certain parameter sequences are likely to result in superior assembly performances. However, these works per se do not address spatial reasoning or cooperation between multiple robots in assembly tasks and the models proposed therein make no claims as computational instantiations of any cognitive phenomena like body schemas or peripersonal spaces.

Separately, computational models for peripersonal (and task-related) space representation of robots have been proposed in literature and used in the context of primitive actions like reaching [29–32] and object recognition [33, 34]. Recent years have also seen the emergence of computational models facilitating human-robot interactive manipulation tasks ranging from simple ones—dealing with ‘give, show, make accessible’ to the cooperating human—to more advanced models that involve proactive task selection and cooperation [35, 36]. Traditionally, these tasks have been considered as spatial problems solved by robots using sophisticated geometric rotation or line-of-sight computation to infer what their human partner could see in a scene [35] and thus engage in simple cooperative actions.

In contrast to direct geometric processing of perceptual and spatial information for reasoning and inference, the proposed framework looks at *learning and formation of internal models instead*. This approach is motivated by evidence from neurosciences related to embodied simulation and prediction [37–40] in particular:

- *Simulation of action*: We can activate motor structures of the brain in a way that resembles activity during a normal action but does not cause any overt movement [39, 41];
- *Simulation of perception*: Imagining perceiving something is actually similar to perceiving it in reality, only difference being that the perceptual activity is generated by the brain itself rather than by external stimuli [42, 43];
- *Anticipation*: There exist associative mechanisms that enable both behavioural and perceptual activity to elicit other perceptual activity in the sensory areas of the brain. Most importantly, a simulated action can elicit perceptual activity that resembles the activity that would have occurred if the action had actually been performed [38, 44].

Guided by these studies, we describe a bioinspired neural architecture for goal-directed reasoning and cooperation between multiple robots based on the coupled interactions between the internal models representing the robot’s body and its peripersonal space. These two interacting internal models engage in a range of anticipations related to the feasibility and consequences of the actions of oneself and the interacting partner to allow planning in a complex environment in the context of a joint goal. Both the internal models (of the body and its peripersonal space) are jointly learnt through a process of sensorimotor exploration. The neuroscientific perspective — that internal models of both the environment and the body are intertwined to synergistically interact in goal-directed behaviours and hence they should be developed in parallel [38, 45, 46] — further substantiates our modelling approach. The proposed neural framework exploits several well-grounded computational concepts in literature mainly,

- the idea of growing neural gas [47] (an extension of self-organizing maps [48]) for internal representation of the peripersonal space;
- neural field dynamics [49, 50] to organize goal-directed reasoning and cooperative behaviour in shared spaces;
- the idea of reward fields [51] for reasoning about action sequences based on the unfolding spatiotemporal dynamics of the task;
- learning of such behaviour-modulating reward fields over experience gained through exploration [52];
- the idea of passive motion paradigm (PMP) [53–55] based on the notions of Equilibrium Point Hypothesis [56, 57], internal simulation theory [58] and synergy formation [59] for internal representation of the agent bodies for simulation, reasoning and execution of goal-directed actions.

While some interesting internal models of the body have been proposed in the literature [60, 61], these models have neither been applied to any complex planning tasks such as assembly nor applied in complex industrial environments. The architecture presented here exploits the internal models of body and space, building up on [51, 52], by hypothesizing their synergistic interaction on multiple embodiments for reasoning and goal-oriented cooperation in complex real-world industrial assembly tasks.

### Contributions of This Work

The main contributions of the work described in this article are:

- A *neural* framework for *joint learning* of *internal models of the body and the peripersonal space* for any robotic embodiment though *sensorimotor exploration*.
- A reward-based dynamics for the proposed framework that *facilitates a range of anticipations related to spatial planning and cooperation in a goal-directed manner*.
- *Implementation of the framework* developed in (a and b) on two industrial robots operating in a shared workspace in a *real-world industrial assembly line*, *performing complex joint assemblies.*
- *Benchmarking* of the proposed neural framework *against a typical industrial solution* designed for the same assembly task to evaluate success performance.

Additionally, open source code for learning and use of these *task agnostic* internal models along with necessary documentation is made available online (see the Supplementary Information).

The remainder of the paper is organized as follows: the ‘The Robots and the Experimental Setup’ section gives a description of the employed robots and their environment as well as outlines the assembly task. In ‘The Computational Framework’ section, we present the internal models for body and task-space of the robots. We specially focus on two key aspects of the internal models: (a) their acquisition through exploration by the two robots discussed in ‘Internal Body Model’ and ‘Internal Model for Peripersonal Space Representation’ sections, and (b) their coupled interaction with a reward field dynamics that generates goal-directed behaviour, discussed in a following section. In the ‘Goal-Directed Spatial Reasoning for Joint Operation in Shared Workspace’ section, we report results of robots performing in parallel an industrial assembly task in unstructured environmental scenarios. In the ‘Cooperation Between the Robots to Achieve Otherwise Unrealizable Goals’ section, we show how the two robots use the proposed framework to internally simulate sequences of actions and operate jointly in a purposeful manner to realize an otherwise unrealizable assembly task. The last section concludes the paper.

### The Robots and the Experimental Setup

**Fig. 1:**
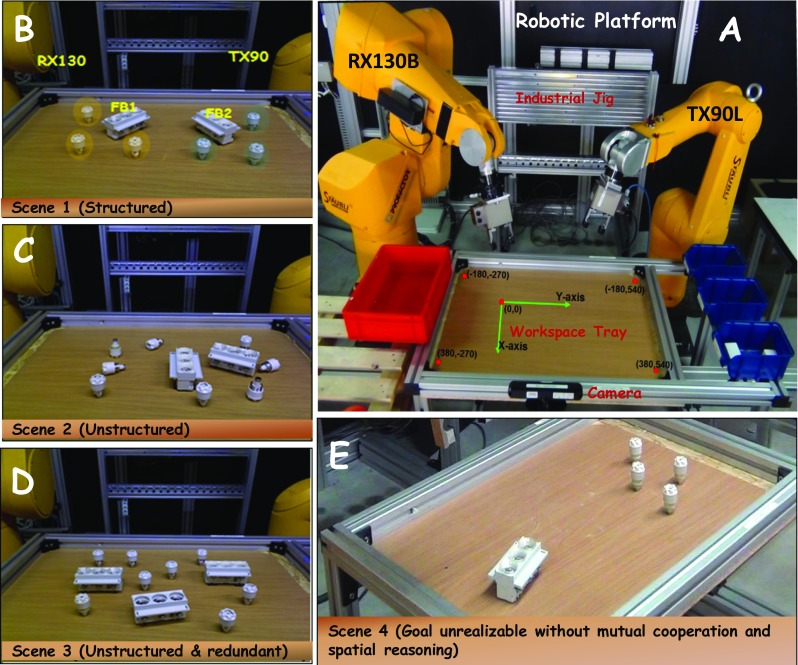
**a** The robotics platform used for experiments with two robots, the camera and the workspace tray. **b**–**e** The typically different possible setups in which objects can be lying in the workspace. **b** A structured scenario where a fuse-box with three fuses is near each robot and parallel assembly can be easily realized. **c** An unstructured setup where objects are lying in random places and reasoning is essential for successful joint assembly. **d** An unstructured and redundant setup where there are more than two fuse-boxes to work with. **e** A scenario where none of the two robots can reach all the desired objects, so the assembly is not realizable in a direct way without mutual cooperation and spatial reasoning

The neural framework for reasoning and cooperation presented in this paper is implemented on an industrial platform to perform assembly tasks. Figure 1a gives an overview of the set up on which experiments were carried out. This robotic platform consists of two industrial manipulators, namely the Stäubli [62] RX130B and TX90L robots each with 6 degrees of freedom providing high flexibility and precision. The platform includes a servo-electric two-finger parallel gripper from SCHUNK PowerCube [63], one for each robot. The manufacturer-offered PowerCube API library provides functions to control the grippers. The platform is augmented with a Kinect PrimeSense camera for visual perception. The camera is the only source of external sensory input to the robots. Visual perception is structured around two major components. The first of these components is concerned with the detection of objects [64] of interest in RGB-D images that have been captured with the Kinect sensor. Following the detection process, location of the detected objects in space is estimated using a 3D pose estimation method from Lourakis and Zabulis [65]. The industrial setup has a workspace tray mounted in front of the two robots on which objects for assembly are arbitrarily placed (Fig. 1b–e). Both the robots and the camera are calibrated to the same frame of reference with the origin lying on the surface of the workspace tray (see Fig. 1a). The workspace is limited in X and Y directions to the area visible to the camera, that is an effective volume of 700 × 800 × 350 mm^3^. Standard objects used in industrial assembly like fuses and fuse-box stands were used for all experiments (see Fig. 1b–e).

#### Assembly Task

Fuses and fuse-box stands are scattered around in the workspace and the robots have to complete maximum number of possible fuse-box assemblies (i.e. insert fuses into fuse-stands). Figure 1b–e shows various possible types of scenarios that robots have to deal with. Appropriate action sequences must be generated by both robots based on the setup of objects in the workspace to compete assemblies quickly and successfully while operating in parallel. As an example, scenario shown in Fig. 1b is well-structured to allow *spontaneous and collision-free operation* of both robots during assembly process (for example: the RX robot assembling one fuse-box (FB1) and the TX robot assembling the other fuse-box (FB2)). In the scenario shown in Fig. 1c, objects are scattered arbitrarily in the workspace. Hence, planning which robot acts on which object (at different time instances) is necessary to realize multiple joint assemblies by the robots. Acting without reasoning will result in collisions between the robots, collision with the objects, and time-consuming serial assembly sequence. The scene depicted in Fig. 1d is both unstructured and redundant (more fuse-box holes than fuses) requiring further reasoning about which two of the three fuse-boxes to work with. Figure 1e shows an interesting scenario where the assembly is not directly realizable by either of the two robots since all the desired objects for a complete assembly are not reachable (for example, fuse-box stand is not reachable to the TX robot and fuses are not reachable to the RX robot). In general, based on the spatial configuration, appropriate action sequences must be generated by both robots to operate in parallel (with collision avoidance) and realize assemblies successfully.

In this context, we describe a bioinspired neural architecture for goal-directed cooperation based on the coupled interactions between multiple internal models, primarily the robots’ bodies and their peripersonal spaces. The internal models of each robot’s body and its peripersonal space are learnt jointly through a process of sensorimotor exploration and then used to engage in a range of anticipations related to the feasibility and consequence of potential actions of oneself and the other (in the context of a joint assembly). In the following sections, we describe how these internal models are learnt and applied in real-world setups for an efficient parallel assembly of industrial objects by two robots.

## The Computational Framework

**Fig. 2:**
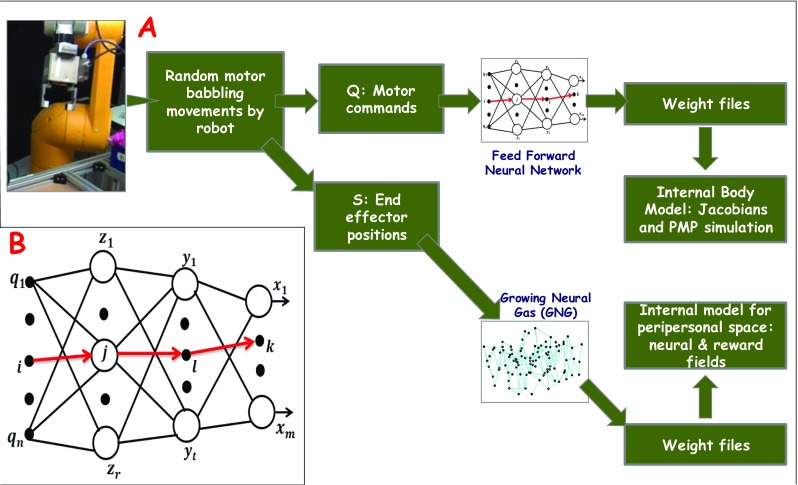
**a** This block diagram shows the stage of data generation by a robot followed by learning of the two internal models. Random babbling movements of the robot in the workspace give rise to two data sets: one of joint rotation readings and the other set of corresponding coordinates of the end-effector. A standard feedforward network learns the mapping between the intrinsic and extrinsic spaces using error-backpropagation. The Jacobian is then extracted from the learnt connectivity matrices (weight files) which represents the internal body model for action simulation and execution. At the same time, a growing neural gas is used to learn the topology of the peripersonal space using the set of end-effector coordinates generated during babbling. The learnt GNG network is stored as connectivity matrix and sensory weight matrix (weight files) to serve as the representation of the peripersonal space of a robot. **b** A feed forward neural network composed of an input layer {*q*_*i*_}, two hidden layers {*z*_*j*_} and {*y*_*l*_} and an output layer {*x*_*k*_}. See the text for details

The following two sub-sections summarize the two internal models that are jointly learnt and form the core of the proposed architecture: (1) internal model of the body and (2) internal model of the peripersonal space. An important feature of the architecture is that both these models are learnt from the same data generated during sensorimotor exploration of a robot in its reachable workspace through a process of random motor babbling. This is outlined in Fig. 2a which shows diagrammatically the main stages of the learning process of the two internal models. Finally, the third sub-section describes how the coupled interaction between these internal models provides us with a framework for spatial reasoning in a parallel assembly task.

### Internal Body Model

The internal model of the body is based on the motor control theory known as passive motion paradigm (PMP) [53–55, 66] that draws on prominent ideas like the synergy formation [59] and the Equilibrium Point Hypothesis [67, 68]. PMP offers a shared computational basis for simulation and generation of action in articulated structures of arbitrary complexity and redundancy. Intuitively, the idea is that given a target for robot end-effector to reach, the process to determine the distribution of work across its joints can be represented as an ‘internal simulation on the body model’. The internal simulation calculates how much each joint would move if an externally induced force (i.e. the goal) pulls the end-effector by a small amount towards the target. This process of relaxation is like coordinating the movements of a puppet by means of attached strings: as the puppeteer pulls the task relevant tip of the body to a target, the rest of its body elastically reconfigures to allow the tip to reach the target. PMP can be defined computationally in the following steps:

1. *Given a target, generate a target-centred virtual force field in the extrinsic space*:

$$ F={K}_{\mathrm{ext}}\left({s}_{\mathrm{T}}-s\right) $$

Where *s*_T_ is the target, *s* is the current position of the end-effector and *K*_ext_ is the virtual stiffness of the attractive field in the extrinsic space. *K*_ext_ determines the shape and intensity of the force field. In the simplest case, *K*_ext_ is proportional to the identity matrix and this corresponds to an isotropic field, converging to the goal target along straight flow lines.

(2) *Map the force field from the extrinsic space into virtual torque field in the intrinsic space*:

$$ T=J{(q)}^T\ F $$

where *J*(*q*)^*T*^ is the transposed Jacobian matrix which is always well-defined. In the next section, we show how these Jacobians can be derived from a learnt internal body model.

(3) *Relax the arm configuration to the applied field*:

$$ \dot{q}={A}_{\mathrm{int}}\bullet T $$

Where *A*_int_ is the virtual admittance (or joint compliance) matrix in the intrinsic space that leads to the distribution of the torques among the joint rotations. In the simplest case, it is an identity matrix.

(4) *Map the arm movement into the extrinsic workspace*:

$$ \dot{s} = J(q)\bullet \dot{q} $$

(5) *Integrate over time until equilibrium*:

$$ s(t)={\int}_{t_0}^t\ J(q)\dot{q} d\tau $$

The last step integration gives us a trajectory with the equilibrium configuration *s*(*t*) defining the final position of the robot in the extrinsic space.

To put in words, at each time step of this cyclic computational process, target goal *x*_*T*_ in the extrinsic space induces virtual disturbance forces *F* on the end-effector, which are modulated by the virtual stiffness *K*_ext_. These forces are then mapped into equivalent torques *T*; this projection is implemented by the transpose Jacobian *J*(*q*)^*T*^. These virtual torques *T* cause incremental joint rotations $\dot{q}$ as allowed by the compliances *A*_int_ of different joints. The incremental change in joint space $\dot{q}$ is mapped to the extrinsic space $\dot{x\ }$ using the Jacobian matrix *J*(*q*), causing a small displacement of the end-effector towards the intended target. This process progresses cyclically till the time the algorithm converges to an equilibrium state reaching the target. While in depth discussions on PMP and its extensions and implementations on various robotic platforms are available in [55, 69] and [66, 70], here, we briefly highlight some key advantages of the PMP formulation against the traditional analytical approaches in robotics:

1. There is no kinematic inversion or cost function optimization since all computations are well posed (one-to-one). Hence, PMP is computationally inexpensive especially while coordinating highly redundant robots. In this sense, PMP is closely linked to other approaches based on active inference [71] that also avoid inverse kinematics. Two recent reviews discuss the pros and cons of these approaches in detail [72, 73].
2. A solution is guaranteed and there are no singularities. This applies even in cases where the target is unreachable. In such a case, the final solution or the output of the forward model (i.e. the end-effector location) provides useful geometric information to trigger further reasoning; such as the desired length of a tool to reach the target.
3. PMP offer runtime configurability. PMP networks can be assembled on the fly based on the nature of the motor task and the body chains or tools chosen for execution.

In the context of this article, what is important is the fact that PMP is not only able to generate real actions but also simulate imaginary movements predicting the sensory consequences of the imagined actions. This is very consistent with the recent research confirming common underpinnings for real and imagined actions [39, 72]. In our view, the internal body model acts as a *link or the middleware* between the real and imagined actions. Running internal simulations on an interconnected set of neuronal networks must be the main function of the body schema in humans. We believe the proposed PMP model provides a possible computational formulation to explain the results from neuroscience. Further, PMP augments the idea that cognitive motor processes such as action planning shares the same representations with motor execution [58]. This allows a cognitive agent to reason about and plan its actions in the environment beforehand in a goal-directed fashion [37, 38, 74]. In this sense, PMP framework closely resonates with the embodied simulation hypothesis discussed in the Introduction.

#### Acquisition of the Internal Body Model

The body schema representation is acquired using random motor babbling movements of a robotic arm in its peripersonal space while the end-effector location is tracked through visual perception. This gives rise to sensorimotor data: a *training set* of joint rotation readings with the corresponding coordinates of the end-effector. Using joint angle vectors as input and the corresponding 3D end-effector location vectors as desired output, a feedforward neural network with two hidden layers is trained through backpropagation of error. Thereafter, the Jacobian can be recovered from the weights of the trained neural network: that represents the body schema, mapping the extrinsic to the intrinsic spaces. Below we discuss the process of acquisition of the Jacobian matrix *J*(*q*).

Let a vector ***q*** represent the state of a robot in the intrinsic joint space for a given pose and a vector ***s*** identifies the position of the end-effector of the robot in the extrinsic workspace for that pose. Then the kinematic transformation ***s*** = *f*(***q***) can be expressed as: $\dot{\boldsymbol{s}} = J\left(\boldsymbol{q}\right)\bullet \dot{\boldsymbol{q}}$ where *J*(***q***) is the Jacobian matrix of the transformation. We train a multilayer feed forward neural network (see Fig. 2b) with two hidden layers to learn the mapping ***s*** = *f*(***q***) where ***q*** = {*q*_*i*_} is the input vector (of joint angles) and ***s*** = {*s*_*k*_} is the output vector (representing 3D position/orientation of the end-effector). ***z*** = {*z*_*j*_} and ***y*** = {*y*_*l*_} vectors are the output of first and second hidden layer units respectively. Equation 1 expresses the mapping, where *Ω* = {*ω*_*ij*_} are connection weights from the input layer to first hidden layer, *O* = {*o*_*jl*_} are the connection weights between the hidden layers, *W* = {*w*_*lk*_} are the connection weights from the second hidden layer to the output layer, ***h*** = {*h*_*j*_} are the net inputs to the neurons of the first hidden layer and ***p*** = {*p*_*l*_} are net inputs to the second hidden layer. Neurons in the two hidden layers fire using the activation function *g* which represents the hyperbolic tangent function tanh(); the output layer neurons are linear.

1 $$ \boldsymbol{s}=f\left(\boldsymbol{q}\right)\Rightarrow \left\{\begin{array}{c}\genfrac{}{}{0pt}{}{h_j={\sum}_i{\omega}_{ij}\ {q}_i}{z_j=g\left({h}_j\right)}\\ {}\genfrac{}{}{0pt}{}{p_l={\sum}_j{o}_{jl}\ {z}_j}{y_l=g\left({p}_l\right)}\\ {}\genfrac{}{}{0pt}{}{s_k={\sum}_l{w}_{lk}\ {y}_l={\sum}_l{w}_{lk}\bullet g\left({\sum}_j{o}_{jl}\ {z}_j\right)}{\Rightarrow \kern1em {s}_k=\kern0.5em {\sum}_l{w}_{lk}\bullet g\left({\sum}_j{o}_{jl}\bullet g\left({\sum}_i{\omega}_{ij}\ {q}_i\right)\right)\ }\end{array}\right. $$

After training the neural network using sensorimotor data generated by the robot, the Jacobian *J*(***q***) can be extracted from the learnt weight matrices using the *chain rule*, as in the following expression:

2 $$ J\left(\boldsymbol{q}\right)=\frac{\partial {s}_k}{\partial {q}_i}=\sum \limits_l{w}_{lk}\cdot {g}^{-1}\left({p}_l\right)\kern0.5em \sum \limits_j{o}_{jl}\cdot {g}^{-1}\left({h}_j\right)\ {\omega}_{ij} $$

Since each robot has 6 joints, the input vector consisted of 6 values and the target vector consists of the corresponding 3D location. The number of neurons in first and second hidden layers of the neural networks was determined heuristically as 32 and 41, respectively. The neural network was trained using Levenberg–Marquardt algorithm. Over five different training runs of 2000 epochs each, results showed that the network converged very well, with an average root mean square error of the approximator less than 0.04 mm at the test.

#### The Body Model in Goal-Directed Reasoning

The Jacobians extracted from the trained network are inserted at appropriate steps in the above given PMP computational algorithm. The Jacobians provide the PMP dynamics with a manipulable representation of the body; i.e. a body model that can be used as a forward/inverse model for goal-directed simulation and execution of actions in real world. In the context of this work, a robot can then use the acquired internal body model to ‘imagine’/simulate reaching movement to a given target and anticipate if the target is reachable or not without making any real movement. This provides the robot with the information of the feasibility of any planned reaching without entering a risk of unexpected failures to reach. In addition, since the forward kinematics of the internal model of body (neural PMP) computes a movement trajectory from the current to the goal position in the extrinsic space, imagined movements of multiple robots in a shared workspace can be explored for possible collisions between the robots before any execution of real movements. An alternative course of actions can be planned to realize the goal in case collisions between the imagined movement trajectories are detected.

### Internal Model for Peripersonal Space Representation

In this section, we describe the internal model for a sparse representation of the peripersonal space of a robot. Such an internal representation is needed to perform a non-uniform quantization of the peripersonal space, thus simplifying the target selection during the assembly task. This representation of space is learnt using a Growing Neural Gas (GNG) algorithm [47] from the same data used to learn the internal model of body (neural PMP). GNG is an unsupervised incremental network model that can learn important topological relations in a given set of input vectors by means of a simple Hebb-like learning rule. The model continues learning, adding new neurons and connections until a performance criterion is met.

#### Acquisition of the Peripersonal Representation

To begin the learning process, the robot randomly explores different spatial locations in its workspace through motor babbling; these spatial locations/end-effector coordinates *S*^*t*^ form the sensory input to the growing neural gas. The free variables that are learnt in this algorithm are as follows. The size of the resulting matrix is indicated inside the parenthesis.

1. *N*: No. of neurons in the GNG network (*N*).
2. *s*_*i*_: Sensory weights for each neuron (*N* × *D*), these are randomly initialized; *D*: degrees of freedom in the sensory space, which is 3.
3. *error*_*i*_: Local estimate of representational error, i.e. the accumulated difference between the actual perception and the best matching unit in the GNG (*N*). This information is particularly useful for growing the GNG.
4. *Age*_*ij*_: Age of lateral connection for pruning off excess and less valuable lateral connections in the GNG (*N* × *N*). Age of all connections is initialized to zero in the beginning.
5. *W*_*ij*_: Lateral weights (these are edges that encode neighbourhood) (*N* × *N*).

**Fig. 3:**
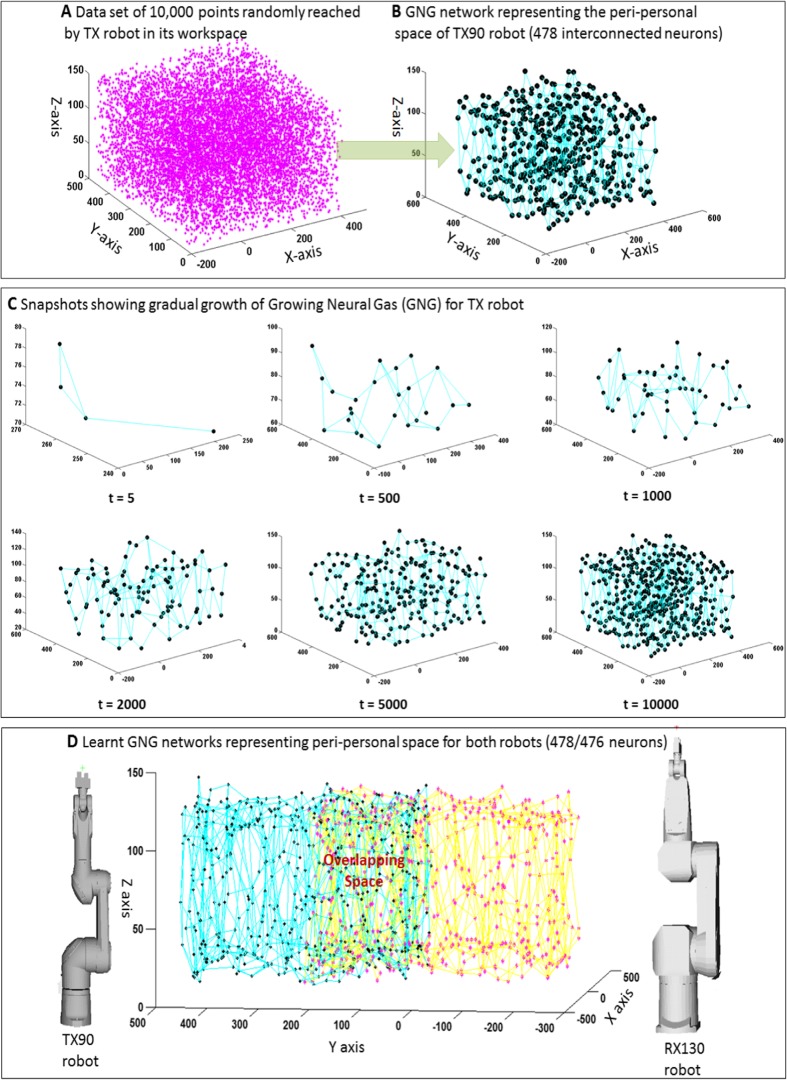
**a** The 10 K location points reached by the TX robot during exploration. **b** The resulting learnt Growing Neural Gas (GNG). **c** The gradual growth of the GNG for the TX robot with increasing exploration in its peripersonal space. In the figure, *t* refers to the number of points in space reached by the robot or iterations of learning. **d** The workspace between the robots as represented by the GNG maps of the two robots. The two networks overlap in the workspace where both robots can *reach* to perform assembly tasks

Below, the algorithm for learning the neural map through randomly generated sequences of sensory *S* data is outlined as a sequence of steps (a–g):

- *Initialization*: Start with one single neuron with randomly initialized sensory weights *s*_*i*_.
- *Acting and observing*: Babble to a random location and acquire the sensory information *S*^*t*^. *t* stands for time or iteration number of the exploration (see Fig. 3 for details).
- *Estimating the winner*: Of all the neurons that exist in the GNG at that point of time, find the neuron ‘*i*’ that shows maximum activity for the observed sensory stimulus *S*^*t*^ at time/iteration *t*. This implies finding the neuron ‘*i*’ that has sensory weights *s*_*i*_ such that ∣|*s*_*i*_ − *S*^*t*^|∣ has the smallest value, among all neurons existing in the GNG at that instance of time.
- *Growing when needed*: New neurons are incorporated into the GNG when the difference between the actual perception and the best matching unit say ‘*i*’ becomes too large. To make this detection more robust, we assume that every neuron in the GNG has a measure of its own local representational error that accumulates with respect to time. For this purpose, we use a low pass filter at a timescale *τ*_*e*_ = 10 as in equation below:

$$ {\tau}_e\dot{error_i}=-{error}_i+\left(1-\frac{1}{\sqrt{2\pi }{\sigma}_e}{e}^{\frac{-{\left({S}_i-S\right)}^2}{2{\sigma}_e^2}}\right) $$

Whenever this error measure exceeds a threshold called vigilance, *error*_*i*_ > *v* (in our case *v* = 0.25), we generate a new neuron *j* with the codebook vector equal to the current perception. Gaussian kernel width *σ*_*e*_ = 1 was used.

(e) *Adapting the sensory weights*: Now adapt the sensory weights of the winner and its topological neighbours (all neurons laterally connected to the winning neuron) by small fractions *e*_*w*_ and *e*_*n*_ of the distance as follows:

$$ {\displaystyle \begin{array}{c}{s}_i\leftarrow {s}_i+{e}_w\left(S-{s}_i\right),\kern0.75em \mathrm{Winner}\kern0.24em i\\ {}{s}_n\leftarrow {s}_n+{e}_n\left(S-{s}_i\right),\forall n\kern0.61em \in \mathrm{Neighbours}(i)\end{array}} $$

*e*_*w*_, *e*_*n*_ ∈ [0, 1]. While setting *e*_*w*_ and *e*_*n*_ too high usually results in an unstable network, with nodes moving all around all the time, setting them too low often makes training slow and ineffective. In all our experiments, we choose the following values: *e*_*w*_ = 0.04 and *e*_*n*_ = 0.0006.

(f) *Adapting the lateral weights*: Lateral weights *W*_*ij*_ are simply edges between neurons that encode neighbourhood and possible state transitions. These links permit spreading of activity in the direction of the gradient of value field and are locally adapted in response to dynamic changes in the world. We employ the simplest mechanism to organize the lateral weights, as proposed by Fritzke [47]. This technique involves growing a lateral connection between successive best winning neurons ^‘^*k*^’^ and ^‘^*i*^’^ with a lateral weight initialized as *W*_*ik*_ = 1, incrementing the age of all other neighbouring lateral connections, and finally pruning off the connections whose age cross an age threshold *Age*_max_ (in our case equal to 25).
(g) *Pruning:* Finally eliminate the dead neurons (with no lateral connections) existing in the system and proceed with the next step of sensory input observation and another incremental phase of learning the free variables in the system using the procedure mentioned above. As newer regions in the workspace are explored, the internal map grows and becomes more densely connected. This process continues till the time the internal map becomes almost quasi stationary.

During this process of training, the robot babbled to about 10,000 locations in its workspace area before settling to a sparse representation of the area explored. Figure 3a shows the generated data points in the workspace of the TX robot and Fig. 3b shows the corresponding learnt GNG network grown to a size of 478 interconnected neurons. Figure 3c shows the topology of the evolving GNG as new incoming sensory information *S*^*t*^ keeps coming or in other words as the robot explores more and more locations in its workspace. Another GNG network representing the peripersonal space of the RX robot is learnt using the same technique. In our results, the GNG network for RX robot grew to a size of 476 neurons. Figure 3d shows the overall structure of the workspace with the two robots and the two GNG networks representing their peripersonal spaces. There is an overlap between the networks representing the intersecting peripersonal areas where both the robots can operate. Before we go into the next section that describes how this representational scheme can be exploited to serve as a general substrate for realizing goal-directed planning and cooperation, below we highlight the relevance of the proposed approach from a neuroscientific perspective.

In humans, peripersonal space representations are pivotal in the sensory guidance of motor behaviour, allowing us to interact with objects and with other people in the space around us [75–77]. It is widely argued that body schema and peripersonal space representations share underlying brain networks. Recent studies have provided evidence that perceiving objects in peripersonal space activates a set of interconnected parietal and frontal areas overlapping with the set subtending voluntary motor action and motor imagery [75, 76, 78]. This trend of research was one of the key motivations to our work for designing a system with intertwined models for body schema and peripersonal space. Here, we emphasize that our model training approach is also very consistent with the neuroscientific perspective that the representations or internal models of both the space and the body are deeply intertwined [45] and synergistically interact to facilitate goal-directed behaviour, and hence, they should be developed in parallel [38, 46]. Furthermore, neuropsychological studies show that brain constructs rapidly modifiable representations of space, centred on different body parts (i.e. hand-centred, head-centred, and trunk-centred). The size of these peripersonal spaces also varies for different stimulated body parts [79]. There is also convincing evidence to show that peripersonal space processing operates in a very plastic and dynamic manner, e.g. peripersonal space of arms is extended due to tool-use [75] and gets shrunk due to amputations [80]. Relating to our case, the industrial robots TX and RX are not identical (TX is a smaller robot), and for many other tasks they are used separately, sometimes extended with tools coupled to their grippers. Hence, we deploy separate GNGs for the two different robots.

#### Spatiotemporal Reward Field Dynamics for Goal-Directed Reasoning

Now that each robot has a representation of its workspace, we discuss the mechanisms to organize the action sequence of the robots in a goal-oriented way. By providing each robot with information on what object to act on and when, the two robots can complete the assembly task successfully. An optimal action sequence will maximize parallel operation of the two robots during assembly and minimize involvement of any other mechanisms needed for collision avoidance which delay the assembly process.

To organize goal-oriented assembly, a reward-based neural field is applied to the GNG network on top of the sensory input driven neural field in the network. The spatiotemporal dynamics of the reward field organizes the sequence of the actions taken by each robot by prioritizing execution of actions which fetch maximum reward. Such a reward field can be based on a default plan [51, 52] or can be learnt by robots through exploration and experience (see the Supplementary Information). The layout of the workspace in our experimental setup is such that the position of the robots is roughly symmetric along the *y*-axis of the workspace. Therefore, a good strategy is to structure the instantaneous reward gained by a robot when acting on an object as a function of the distance (in *y*-axis only) of the object from the robot. This would imply for a robot to minimize entering the overlapping workspace (see Fig. 3c) and to keep within its unshared workspace for as many successful assemblies as possible. This default reward *R*_*i*_ associated with a neuron *i* is given by

$$ {R}_i=\frac{1}{Z}\left({e}^{\frac{-{\left({y}_i-Y\right)}^2}{2{\sigma}_R^2}}+{R}_c\right) $$

Here, *y*_*i*_ denotes the *y*-value of the sensory weight of *i*th neuron and *Y* is the *y*-coordinate of the location of robot with respect to origin. *R*_*c*_ (=1, in our experiments) is a constant minimum reward to a robot for merely acting in the world. *Z* is chosen such that

$$ \sum \limits_i{R}_i=1 $$

**Fig. 4:**
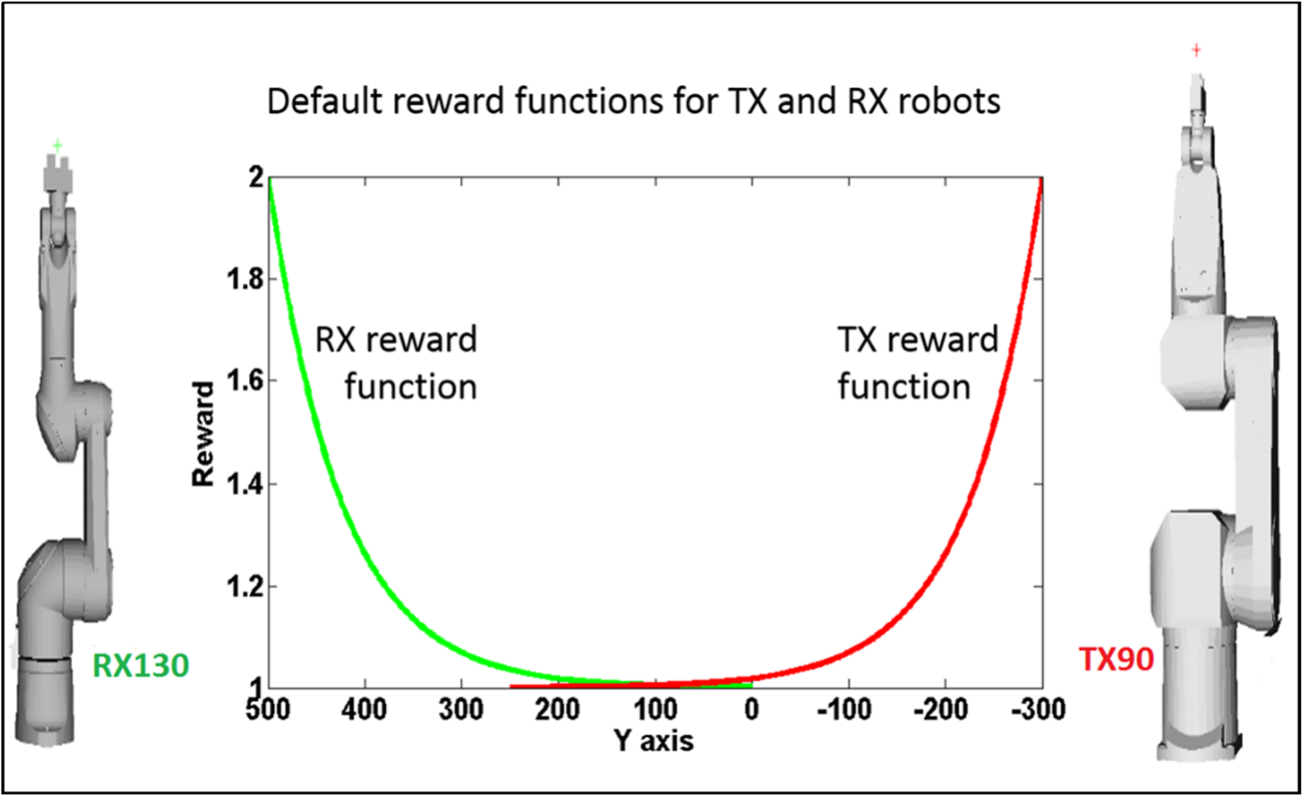
The structure of the default reward functions used for the two robots

This reward function based field dynamics will elicit maximum rewards for objects that are minimally distant from the robot along the *y*-axis. Figure 4 shows the resulting reward functions for the two robots that we used in all real-world assembly tasks. From this graph, it is easy to conclude that the spatiotemporal dynamics of the reward structure will enforce each robot to prioritize acting on objects which are minimally distant from the robot in their *y*-axis.

#### Localization of an Empty Area in the Workspace Using the Internal Model for Peripersonal Space

In response to a sensory input *S*, the activity *x*_*i*_ of a neuron in the GNG network can be given by a Gaussian kernel which compares the sensory weight *s*_*i*_ of a neuron *i* with the current perceptual input *S*

$$ {x}_i=\frac{1}{\surd 2\pi {\sigma}_s}{e}^{-{\left({s}_i-S\right)}^2/2{\sigma}_s^2} $$

Clearly, for any sensory input (which is the 3D location (*X*, *Y*, *Z*) of the centroid of any object in the scene), a neuron which has internal sensory weights closest to the incoming signal will exhibit the highest level of activity in the network and thus best represents the input. For any given spatial layout with *n* objects in a robot’s workspace, *n* neurons of the corresponding robot’s GNG network will show maximal activations corresponding to the locations of the objects in the scene. Neurons nearby these ‘most active neurons’ with also elicit higher activity than the rest of the neurons in the network. Thus, regions in the network where neurons are more active than others represent the occupied areas in the workspace where as regions which show least activity are empty areas in the workspace of the robot.

During cooperative behaviour (see the ‘Cooperation Between the Robots To Achieve Otherwise Unrealizable Goals’ section), a robot may need to move objects from its own non-shared workspace into the shared workspace for the other robot to work with. Since the shared workspace can be cluttered with objects, the spatial reasoning system must find an *empty* area in the shared workspace for the robot to place down an object (fuse). This is to avoid collision of the robot gripper with objects in the scene. Using the spatial configuration as the sensory input to the internal model of a robot’s peripersonal space, we look at the activity of all the neurons in the GNG network representing the shared workspace. The activity of each neuron is a result of the cumulative influence of sensory inputs that fall within a threshold distance to the neuron’s weights. In our experiments, the threshold distance is equal to the width of the robot’s gripper. The neuron which shows least activity has its weights most distant from other objects lying within the threshold distance. We look for a neuron *i* with an activity *x*_*i*_

$$ {x}_i=\min \sum \limits_T\frac{1}{\surd 2\pi {\sigma}_s}{e}^{-{\left({s}_i-{S}_j\right)}^2/2{\sigma}_s^2} $$

where *S*_*j*_ is the 3D location of every object that lies within the distance threshold *T* from the neuron’s sensory weights *s*_*i*_. The area centred at the location of this neuron is an empty area where the object can be safely placed down without colliding with other objects in the workspace. Later in the ‘Cooperation Between the Robots To Achieve Otherwise Unrealizable Goals’ section, we show a case in which empty areas of specific radii are localized and exploited for cooperative behaviour between robots in situations where a single robot cannot realize an assembly task on its own.

### Coupled Interaction Between Internal Models of Body and Space

**Fig. 5:**
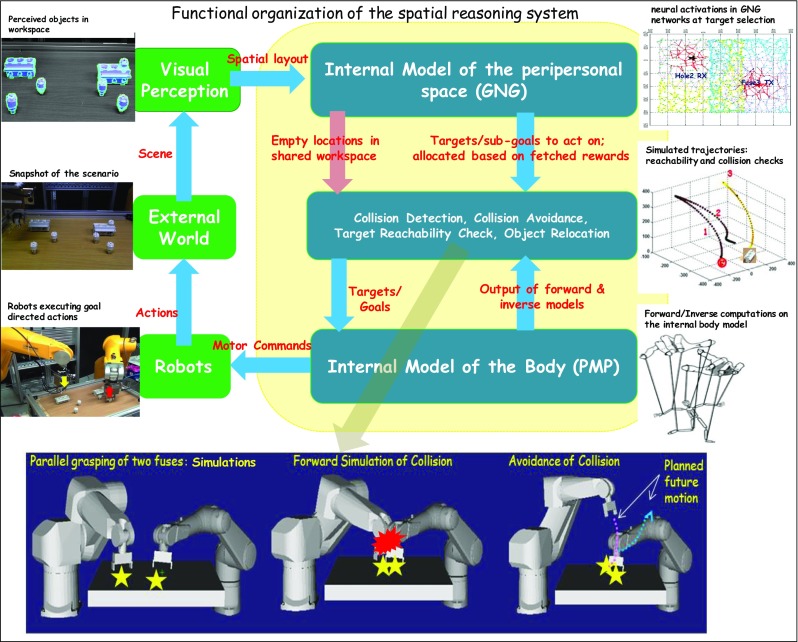
The overall spatial reasoning system with the building blocks and the flow of information between them. An external world scenario of the workspace is passed to a visual perception system that detects the objects and their locations in the scene. This spatial configuration information is forwarded to the internal model for peripersonal space representation which based on the reward field dynamics chooses object(s) to work on and allocates them as targets to the internal body model. The internal body model performs action simulations generating motion trajectories. These trajectories are evaluated for possible collisions. Synthesized motor commands (from the body model) are forwarded to robots to execute movements in parallel in case no collisions are detected, otherwise in serial. The bottom panel shows some simulated results of parallel operation of both robots without collision, anticipated collision and re-planned motion after collision is detected

The problem of two robots performing parallel assemblies in shared workspaces is challenging both from the perspective of reasoning and that of control as well. To plan action sequences based on what objects to act on and when for *time efficient* assembly; and to work *safely and robustly* requires multiple subsystems providing different sub-functionalities to be coherently integrated. In the following lines, we give a description of the overall mechanism conceived to deal with the multi-faceted problem of efficient parallel assembly described in the ‘The Robots and the Experimental Setup’ section. The developed mechanism is a result of synergistic interaction between multiple subsystems (discussed in ‘The Computational Framework’ section) working together. Figure 5 depicts a block diagram of the main components of the reasoning system and the flow of information between these subsystems. Below we outline the functionalities provided by these subsystems and their coupled interactions for realizing the assembly task:

1. Peripersonal space models with reward dynamics: These GNG models account for representation of (1) reachable workspace for a robot with a reward structure that evolves over the course of assembly process as the scenario changes; (2) shared workspace between the robots; and (3) empty and occupied regions in the peripersonal spaces of the two robots. These representations work as resource allocators as described in the ‘Internal Model for Peripersonal Space Representation’ section. Based on the spatial configuration, these space representations allocate sub-goals (fuses and holes) to both the robots using the reward field dynamics. From the spatial layout of the scene as perceived by the visual system, the configuration of the scene (i.e. different objects and their 3D locations in the workspace) generates neural field activity in the two GNG models. In other words, the neural activity in these GNG networks is an internal representation of the spatial layout of the scene outside. Now, because of the corresponding reward structures imposed upon the two GNG networks, each GNG network elicits highest reward for a particular object. Objects fetching highest rewards become targets and their locations are forwarded to the internal model for body. Target selection occurs in two steps: A robot’s GNG selects a target fuse followed by a target hole whenever available.

2) Internal models of the body for simulated and real actions: Given the reachable target goals for the two robots, the PMP-based internal body models simulate solutions in the joint space of the robots (motion trajectories) to reach the targets.

*Collision detection* follows. From the anticipated motion trajectories, possible collisions during parallel execution of these simulated trajectories are estimated. To implement this, the 3D locations of the two distal joints (nearest to the end-effector) of both the robots are computed at multiple points along the two anticipated motion trajectories. The 3D locations are calculated using forward kinematics of the internal models. If the calculated distance between the joints in the extrinsic space is below a threshold value any time during simulated motion, a possible collision is detected. In case no collision is detected, synthesized motor commands corresponding to the two trajectories are forwarded to robots to operate in the workspace in parallel. As for example, the bottom left panel of Fig. 5 shows two robots performing different sub-tasks in simulation (e.g. grasping two different fuses).

Objects in the peripheries of the overall workspace are assembled first. However, as the scenario evolves during the process of assembly, the spatial layout and hence the reward structure pushes the two robots to operate towards the middle of the overall workspace. The neural activity fields in the two GNG networks with reward dynamics select increasingly closely lying targets. Hence, despite reward-efficient allocation of sub-goals by the internal models for peripersonal spaces, collisions are possible in the shared workspace. Whenever possible collisions are detected from the anticipated motion trajectories as described above (see for example a simulation result in Fig. 5 bottom middle panel), movements of the two robots need to be re-planned to avoid collisions. *Collison avoidance* is implemented by serializing the two robot actions one after the other. This occurs by alternately allowing one of the robots to complete its movement (a sub-task) while keeping the other robot away from the shared workspace area at an initialized location (Fig. 5 bottom right panel). A sub-task completion is followed by moving the robot to an initialization position and allowing the other robot to complete its own sub-task.

However, in case a robot’s internal peripersonal space model selected a fuse but cannot find a reachable fuse-box hole in the next target selection step, the model selects an empty location in the shared workspace for the robot to place the fuse. After this *object relocation*, another robot will find both the fuse and fuse-box reachable and will trigger assembly.

The next section presents the experimental results which demonstrate the coupled interaction between the internal models for realization of the task of parallel assembly.

## Results

### Goal-Directed Spatial Reasoning for Joint Operation in Shared Workspace

**Fig. 6:**
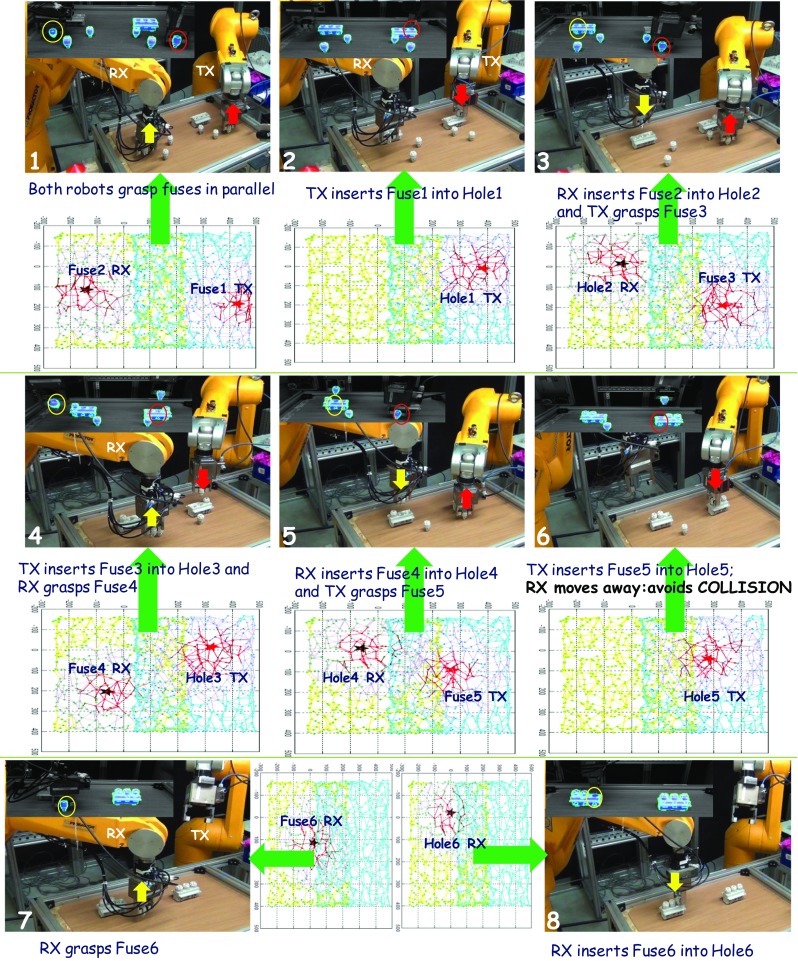
The two industrial robots operating parallelly during an assembly task in a typical unstructured set up. Different panels numbered in a sequence show how 6 fuses lying at various spatial locations are successfully inserted into 6 holes by both robots operating in parallel. See the text for details and refer to the supplementary video (Online Resource 1)

We describe a parallel assembly of fuse-boxes in a typical real-world industrial setting as an example of goal-directed reasoning by the robots employing the interacting internal models. Figure 6 presents a set of panels capturing the behaviour of the two industrial robots in an unstructured setup (i.e. when objects are scattered at random locations), where the goal is to jointly assemble fuse-boxes in the workspace (in the present setup two fuse-box stands and 6 fuses are present). Note that the peripersonal space internal model itself is agnostic to the number of objects or where they are, but will ensure maximum number of assemblies with both the robots working in parallel.

The set of panels in Fig. 6 is sequentially numbered through 1 to 8 following the order in which the robots carry out actions to complete the assembly process. Each numbered panel is associated with three snapshots:

- The workspace as detected by the vision system (the top left corner of each panel with objects as blue 3D-meshes). Objects allocated in each assembly sub-task by the peripersonal space models are shown enclosed in yellow circles for the RX robot (on the left) and red circles for the TX robot (on the right);
- A view of the robots in action, the main panels. These panels are numbered from 1 to 8 in the sequence in which the assembly task unfolds. Up arrows and down arrows depict respectively the ‘grasp-pickup’ and the ‘insert’ operations of the two robots (coloured yellow for the RX robot and red for the TX robot);
- A top view of the state of activity in the RX and the TX peripersonal GNG networks corresponding to the status of target selection progress in the assembly task (below each numbered robot view panel).
  Highly rewarding neurons corresponding to new targets (and the neighbouring neurons) are denoted by stars (black in colour for the RX robot and red colour for the TX robot).

At the start of the assembly process, the spatial layout of the scene imposes activity on the two peripersonal GNG networks. The fuse nearest to a robot along y-direction is rewarded the highest. Fuses that fetch maximum reward for each robot elicit highest activity in the corresponding GNG networks (see GNG snapshot in panel 1). These maximally rewarding fuses are forwarded to the two body models of the robots. The internal body models generate joint space solutions producing motion trajectories to their respective targets. Using the forward kinematics of the body models, two motion trajectories are generated in extrinsic space and then analysed for any possible collisions. In this case (panel 1), no collisions between the trajectories are detected. Hence, the body models send the synthesized motor commands to both the TX and the RX robots to grasp the two fuses in parallel. Next, the TX robot receives the first fuse-box hole as a target to insert the grasped fuse (panel 2). The TX body model generates the motion trajectory which is compared to the RX robot’s current motion trajectory and no possible collision is detected. Therefore, the TX robot inserts the fuse and proceeds to grasp the next target fuse allocated. Meanwhile, the RX robot also inserts the first fuse (panel 3). So far, the two fuses are already assembled in parallel, without the need to trigger any action re-planning for collision avoidance. Panel 4 shows the next sequence of actions with the RX robot grasping another fuse while the TX robot inserting the fuse it grasped in panel 3. So far, all the trajectories have been collision free allowing parallel movements. Note that with the targets in the peripheries of the workspace assembled, the GNG neural fields must choose increasingly close-by targets (see panel 5). Thus, the spatial planning for parallel operation is slowly reaching its limits, and further parallel operations will lead to collisions as estimated from the next anticipated motion trajectories. This triggers serialization of the next two movement trajectories to avoid collisions. Hence as seen in panels 6–7, where the TX robot is to insert the fuse 5 and the RX robot is to grasp the fuse 6, collision is detected in the internal simulations of the two trajectories in parallel. Therefore, as the TX robot inserts fuse 5, the RX robot waits outside the workspace till the TX robot completes the insertion and moves away, and then the RX robot approaches fuse 6. Since no more fuses are remaining, the TX robot initializes while the RX robot inserts fuse 6. In this way, six fuses lying at various spatial locations are successfully inserted with both robots operating collision-free in the shared workspace. The internal models for peripersonal space representation keep allocating sub-goals to the two robots at different time instances during the evolution of the parallel assembly, and internal body models keep generating simulated movements to allow detection and avoidance of collisions and real movements for assembly operations. The reader is referred to the supplementary video (Online Resource 1) showing the robots performing parallel assembly as described in this section.

### Cooperation Between the Robots to Achieve Otherwise Unrealizable Goals

**Fig. 7:**
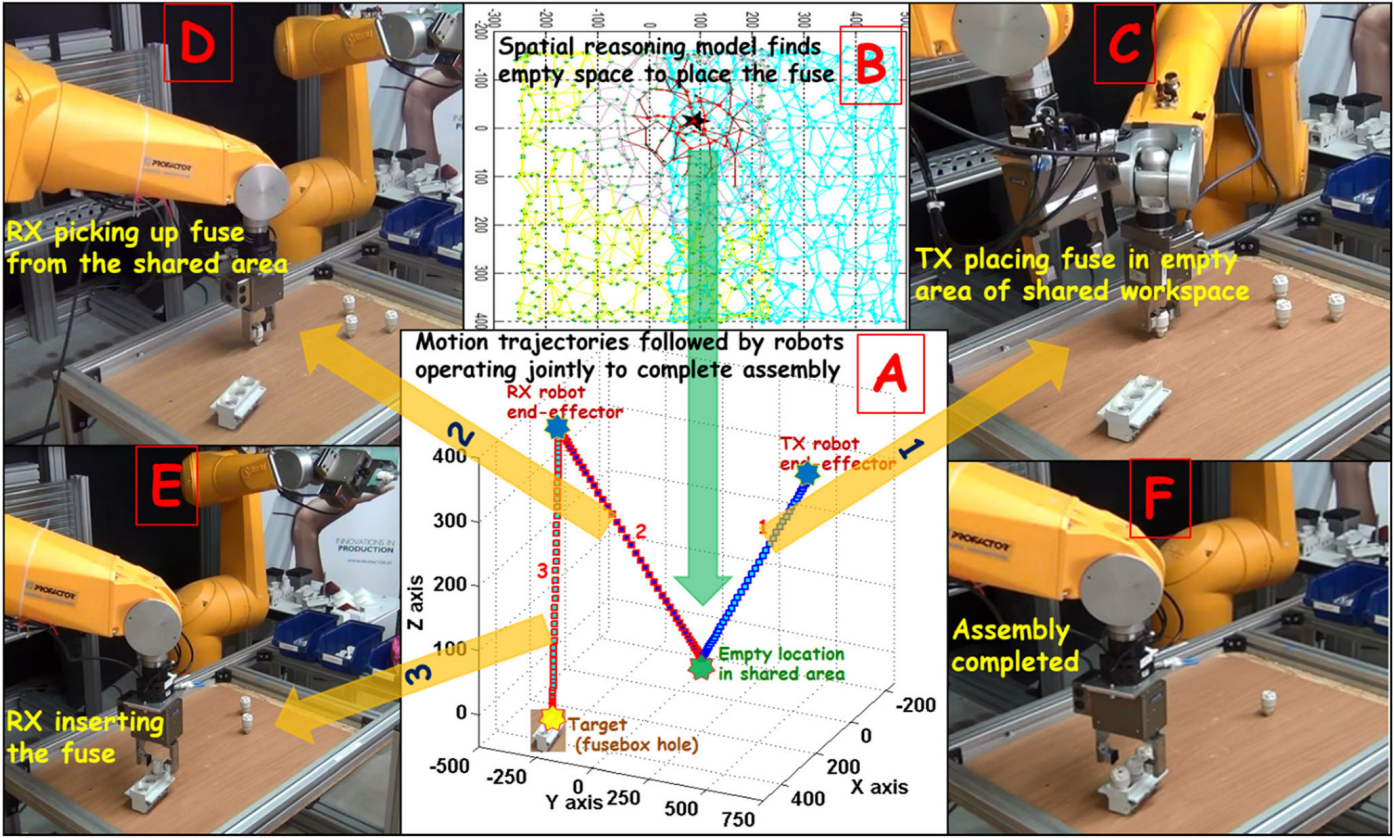
**a** The different motion trajectories followed by the two robots to cooperatively insert a fuse into fuse-box. TX (blue trajectory) places the fuse in the empty area from where RX (red trajectories) picks it up and inserts into fuse-box. **b** The top view of the activity in GNG networks and the minimally activated neuron (in black colour) within a threshold distance (see the ‘Internal Model for Peripersonal Space Representation’ section for mathematical details). The place in the workspace corresponding to the neural weights of this neuron is the empty location (green star) in the shared workspace. **c** The TX robot placing down the fuse at the empty location. The RX robot picks up the fuse (in **d**) from where TX placed it. **e** RX inserting the fuse. **f** The completion of the assembly task by the robots repeating the same sequence of actions till all holes are filled. Refer to the supplementary material (Online Resources 2 and 3) for videos of the assembly using cooperation between robots

In an assembly task like ours, since objects in the workspace are positioned randomly, scenarios can arise where neither of the two robots can perform assembly on its own. As for example in Fig. 7c, where all the fuses in the scene are in the peripersonal space of the TX robot, none of them is reachable to the RX robot and vice versa for the fuse-box stand. In this case, the TX robot can pick up the fuses but cannot reach the fuse-box stand to insert into; similarly, the RX robot cannot reach the fuses in the first place to begin assembly. However, if the robots choose to cooperate in a meaningful way, assembly of the fuse-box can be performed. The idea is that the TX robot can place the fuses at some empty location in the shared workspace reachable to both the robots, from where the RX robot can pick them up and insert them into the fuse-box stand. Here, we describe this process of cooperation using the results shown in Fig. 7.

In the scenario depicted in Fig. 7, as the spatial layout activates the neural fields in the peripersonal space models, only the peripersonal space model (GNG network) for the TX robot finds a target fuse but there is no target fuse detected by the RX robot’s GNG network. Though a fuse-box stand is reachable to the RX robot, it must select a fuse first to start the assembly; therefore, it keeps inspecting its peripersonal space constantly for any fuses. Meanwhile, the target from the TX robot’s GNG network is forwarded to the TX robot’s body model that generates and executes a motion trajectory to grasp the fuse. However, after the TX robot grasps the fuse, its peripersonal space model cannot detect a fuse-box hole as the next target. In this case, the TX robot’s peripersonal space model selects an empty area within the *shared* workspace as a dummy target to place the fuse (see Fig. 7a, b). The shared space boundary limits are applied on the robot’s GNG representation for localization of the empty area. The process of identifying an empty area in the shared workspace is discussed before in the ‘Internal Model for Peripersonal Space Representation’ section. The TX robot places down the fuse at the empty location in the shared workspace (Fig. 7c). In the next step, as the spatial layout activates the GNG network of the RX robot again, a target fuse is found. The fuse is grasped by the RX robot (Fig. 7d). Following this, a target hole is also selected by the RX’s GNG network into which the RX robot inserts the fuse (Fig. 7e). Next, through GNG activations, the TX robot gets another fuse as target. Thereafter, the same sequence of actions as carried out for the first insertion above is repeated for all the fuses until the assembly task is complete (Fig. 7f). In summary, the interactive action sequence during cooperation is: (1) GNG selection of a fuse by TX; (2) PMP grasping of the fuse by TX; (3) GNG selection of an empty area by TX; (4) PMP placing of the fuse in the empty area by TX; (5) GNG selection of the fuse by RX; (6) PMP grasping of the fuse by RX; (7) GNG selection of fuse-box hole by RX; (8) PMP insertion of the fuse into the hole by RX; and (9) repeat steps 1 to 8 until fuse-box assembled.

## Conclusion

Goal-oriented cooperation with other agents in a shared workspace is a ubiquitous aspect of our day-to-day activities and fundamentally requires a synergistic interaction between multiple core subsystems involved in perception, action, learning, prediction and reasoning. In the setting of a real-world industrial assembly scenario where multiple robots are jointly operating in a shared workspace to realize a goal (in this case the assembly of a fuse-box), we described a bioinspired neural architecture for goal-directed cooperation based on the coupled interactions between multiple internal models, primarily of the robots’ body and its peripersonal space. The proposed architecture was evaluated/benchmarked against a state of the art industrial system performing the same assembly task (see the Supplementary Information). The overall performance of the presented architecture was comparable in all aspects against the industrial benchmarking system. To our knowledge, this is one of the first works that employ interacting, learnt internal models of the body and the peripersonal space in complex spatial reasoning assembly tasks. It is also an early attempt to pave way for the application of cognitive modelling approach to industrial environments after a rigorous benchmarking process against typically engineered methods.

The rationale behind the computational framework is guided on one hand by the emerging studies from neurosciences that provide converging evidence related to the existence of internal representations of the body as well as the peripersonal space in the brain [55, 72, 81] and on the other hand emerging evidence in support of the embodied simulation hypothesis towards generation of cognitive behaviour [38, 40]. That humans have an integrated, internal representation of their body is strongly suggested by the variety of pathological conditions which can only be explained by a deficient internal representation [82] or by sensory illusions [83]. Modern neuroscience has greatly enriched the concept, with numerous studies [84] identifying cortical areas in fronto-parietal cortex integrating proprioceptive and exteroceptive sensory information to maintain a coherent /updated internal representation of the spatiotemporal organization of the body. Further, distributed, multi-centred neural activity is consistently detected in the brain during different conditions like imagination of movement (what is doable by oneself), observation/imitation of other’s actions (what the other is doing/can do) and comprehension of language [39–41]. The general insight emerging from this body of literature is that the fundamental problems of shaping motor output during action execution and providing the self with critical information related to feasibility, consequence and understanding of potential actions (of oneself or others) to engage in goal-directed reasoning are closely intertwined. From an evolutionary perspective too, in organisms with complex bodies and inhabiting unstructured environments, actions are *goal-oriented* and not just stimulus oriented, fundamentally requiring *covert simulation* and *overt execution* of action to seamlessly alternate during the maturation of purposive behaviour and social interaction: to maximize success and ensure survival.

**Fig. 8:**
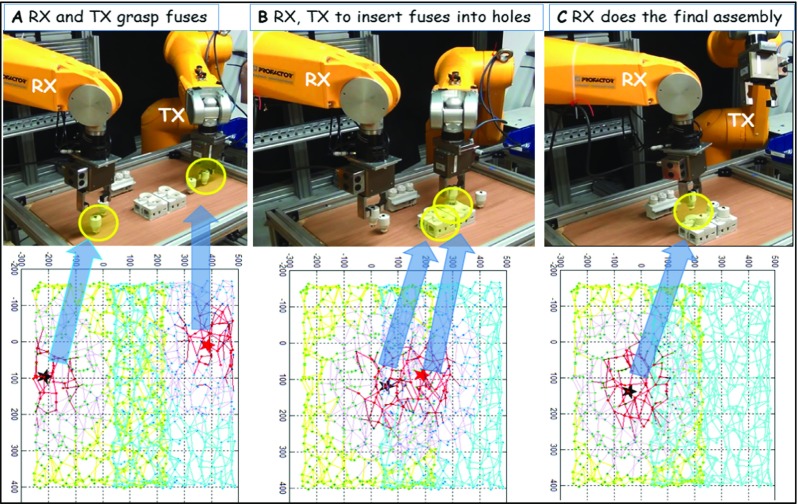
Some of the snapshots of a different assembly scenario where two different types of object sets (fuses and fuse-boxes) are to be assembled. **a** The state where the two robots have assembled one type of fuse-box and have begun to assemble the other type of fuse-box. The top panel shows robots grasping the two fuses allocated based on rewards fetched. The bottom of the panel shows the top view of corresponding neural activations in the internal model representations. **b** The two target holes (yellow circles) allocated to robots for insertion. Given the two holes are close to each other and hence parallel insertion is not possible, TX inserts first and RX waits and can insert only after TX moves away from the workspace. **c** The last scene in the process where RX inserts the last fuse to complete assembly. See the supplementary video (Online Resource 3)

In this context, how multiple internal models (of the body and the peripersonal space) are concurrently learnt by two cooperating robots through a process of sensorimotor exploration and then exploited to engage in a range of anticipations related to the feasibility and consequence of potential actions thereby facilitating joint goal-directed operation was demonstrated in a range of unstructured scenarios. Note that the proposed internal simulation based architecture is domain-agnostic, i.e. independent of the actual task being realized by the two robots in the shared workspace, although in the ‘Results’ section, the behaviours were demonstrated by means of a fuse-box assembly task. This is because the learnt internal models themselves capture the invariant aspects, i.e. the robots own body and its peripersonal space and such a task invariant representation can be easily recycled to reason about any arbitrary joint goals with diverse objects in the shared workspace. Particularly, in an industrial environment, the capability to reconfigure/reuse basic assembly line operation for novel products, with a specified group of cooperating robots in a short duration is a critical desirable feature urgently needing innovative solutions [18]. The bioinspired internal models based neural architecture seamlessly facilitates reuse to new tasks. Figure 8 shows the two robots jointly working in the same work cell in a different assembly scenario, driven by same computational architecture. Given that the internal models are acquired locally by the individual robots, the proposed architecture could be scaled up further with more robotic agents than two as demonstrated in this article and work is presently underway in this direction.

## Electronic Supplementary Material

ESM 1 (DOCX 421 kb)

## Footnotes

1 Cooperation in this article refers to an indirect form of cooperation, akin to *stigmergy* wherein actions of one robot change the environment to enable the other robot to complete an otherwise unrealizable task. This occurs without any direct communication between the two robots.
