# Supplementary material for: Goal-Directed Reasoning and Cooperation in Robots in Shared Workspaces: an Internal Simulation Based Neural Framework
Source: Cognit Comput. 2018 Apr 14;10(4):558–76. doi: 10.1007/s12559-018-9553-1 (PMC6096944; doi:10.1007/s12559-018-9553-1)
Supplement: Supplementary file 1 — (DOCX 421 kb) [file 12559_2018_9553_MOESM1_ESM.docx]

Supplementary Information

to

**Goal-directed reasoning and cooperation in robots in shared workspaces: An internal-simulation based neural framework**

# Performance evaluation of the proposed framework

1. To evaluate the accuracy of the internal body model alone, we conducted an experimental analysis using a simulated environment for both the Stäubli robots. The test involved simple reaching to different target locations in the reachable workspace. For the TX robot, we took an exhaustive set of 24,000 reachable locations distributed uniformly in the workspace. Table 1 gives the statistical details of experimental results for the TX robot. All the values are expressed in millimeters [mm]. The results for TX robot show that the target locations can be reached accurately with a fairly low root mean square error ($<0.04 mm$). The tabulated statistics indicates that the RMSE error and the standard deviation values along z-axis are significantly lower than the corresponding values for x-axis and y-axis. This is because the volume of workspace (about$700\times800\times350 {mm}^{3}$) used to generate the training data is lesser in z-dimension than in the other two dimensions. Owing to a greater density of data points along the z-direction used for training, performance of the learnt body model along the z-axis is better than in the other two axes. A very similar performance table was estimated for the other robot, RX.

| **Statistical Parameter** | **Value** |
| --- | --- |
| Root Mean Square Error | 0.0377 |
| Standard Deviation | 0.0369 |
| Mean Square Error$[\left\vert x \right\vert, \left\vert y \right\vert,\vert z\vert]$ | [0.0512, 0.0381, 0.0142] |
| Standard deviation $[\left\vert x \right\vert, \left\vert y \right\vert,\vert z\vert]$ | [0.0494, 0.0381, 0.0140] |

**Table 1:** Results of the internal body model on TX robot reaching a given target in a simulated environment. All the values are expressed in millimeters [mm].

1. We evaluated the performance of the proposed architecture in the two task scenarios of spatial reasoning and cooperation presented in this paper. We conducted 40 experimental trials of each of these tasks to document the performance of the system in different spatial settings of the assembly task. A complete evaluation and detailed trial by trial analysis of these experiments can be found in a deliverable (D9.5) of the EU FP7 project DARWIN (1). To summarize the relevant results here, we define following success criteria for evaluating performance in key sub-tasks during assembly;

$$Pick Success Rate=\frac{Number of times a fuse is picked up successfully by a robot}{Number of attempts robots make to pick up the fuse}$$

$$Insertion Success Rate=\frac{Number of times a fuse is successfully inserted by a robot}{Number of attempts robots make to insert the fuse}$$

$$Overall Task Success Rate=\frac{Number ofsuccessful fusebox assemblies}{Number of fusebox assembly attempts}$$

Table 2 depicts the evaluation results for 40 different trials of an assembly task requiring spatial planning and collision avoidance between the two robots. It should be noted that these performance rates are equally affected by problems due to vision system like non-detection and mis-localization cumulatively affect the performance of the overall system.

|  | **Pick Success Rate** | **Insertion Success Rate** | **Overall Task Success Rate** |
| --- | --- | --- | --- |
| **Spatial Reasoning** | 89% | 92% | 75% |
| **Cooperation** | 93% | 91% | 85% |

**Table 2:** Performance statistics of the proposed model in an industrial assembly task requiring (a) spatial reasoning, (b) cooperation

Finally, the proposed cognitive system was evaluated/ benchmarked against a state of the art engineered industrial system (1) performing the two versions of the assembly task i.e. spatial planning and cooperation. The engineered benchmark system is described in the next section. Here, we define an evaluation criterion, *Demonstration Specific Success Rate (DSSR)* for spatial reasoning and cooperation tasks separately as following:

$$DSSR (Spatial reasoning )=\frac{Number ofassemblies where no collision between robots occurred}{Total number of assemblies attempted}$$

$$DSSR (Cooperation)=\frac{Number of successful exchange of fuses between robots}{Total number of fuses required that are unreachable}$$

Table 3 summarizes the evaluation results i.e. DSSR for both the proposed internal model based cognitive system and the engineered benchmark system in spatial planning and cooperation tasks over 40 different trials. The overall performance of the presented architecture was comparable in the spatial planning task against the industrial benchmarking system. However, it showed a slightly better performance in its ability to trigger cooperative behaviors between the two robots. Complete trial by trial evaluation and detailed analysis of all the experiments is available in the deliverable (D9.5) of the EU FP7 project DARWIN (1).

|  | **Proposed Cognitive System**  (Demonstration specific success rate) | **Engineered Benchmark System**  (Demonstration specific success rate) |
| --- | --- | --- |
| **Spatial Reasoning** | 100% | 100% |
| **Cooperation** | 88% | 85% |

***Table 3:*** *Performance statistics of the proposed cognitive framework against an Industrial benchmark system for (a) spatial reasoning, and (b) cooperation during an industrial assembly task.*

# The Industrial Benchmark System: Software Architecture

The engineered benchmark system (1) was designed to evaluate the performance of the proposed cognitive framework in comparison to a system based on commercially available software modules. Both the systems were implemented on the same hardware and their performance was compared in the same tasks. We acknowledge the support of PROFACTOR GmbH, Austria that designed the engineered benchmark system. In the following, we give a description of the software components and the overall architecture of the benchmark system used.

The main components of the engineered benchmark system are as follows:

**A. 3D Vision Module:** This module is designed for 3D object recognition and localization based on CAD models of the objects of interest. The method used is based on the concept of feature based matching, which tries to detect correlations of the acquired depth image and pre-processed CAD models of the objects to be detected. This functionality is realized by the Randomized Global Object Localization Algorithm (RANGO) (2), which is an extension of the Random Sampling Consensus Algorithm (RANSAC) (3). The RANGO algorithm is a part of the CANDELOR computer vision library (4), developed by PROFACTOR GmbH and is widely used in industrial and research projects for detecting objects in unstructured environments, e.g. in robot bin-picking applications. The RANGO procedure returns a list of transformation matrices, where each matrix describes the location and orientation of a detected object within the workspace. In addition to object localization, the vision module provides complimentary functionalities listed below.

• Rejection of the false positives detected: Objects “floating” above the table plane and objects whose locations intersect with the table plane are rejected.

• Calculation of potential insertion locations for assembly operations: To detect an empty fuse box hole, the system checks for existing objects around a fuse box’s center holes. If there are less points available than a threshold value, the system tries to transform fuses into the holes, to estimate possible insertion locations.

As a prerequisite, the depth sensor needs to be calibrated relative to the robot workspace, thus sharing the same coordinate system as the two industrial robot systems.

**B. Collision Free Manipulation Planner:** The results of the vision module are used for planning and calculating collision free robot trajectories for grasping and insertion of the detected objects. Based on predefined grasping as well as deposit points on the CAD-Model of the objects, the manipulation planner identifies possible paths in which the object can be grasped. The path planning algorithm is based on the conventional Rapidly-exploring Random Trees (RRT) approach (5,6).

The manipulation planning procedure implemented in the benchmark system can be summarized in the following functionalities.

*Limit Workspace****:*** To enable both the robots to work at the same time a virtual wall, for dividing the workspace into two volumes, is dynamically inserted into the working area. Within the limited workspace, the first robot tries to find a collision free path for grasping and inserting of the object. The first robot is the master and can shift the wall until it finds a valid collision free path. Afterwards, the other robot (slave) may use the remaining workspace for path planning.

*Decide whether the object can be inserted by the grasping robot:* Depending on the selected grip point the manipulation planner component decides whether an insertion path can be directly calculated or a hand-over of the object to the other robot is necessary.

*Plan Handover task:* Depending on the selected grip point, the manipulation planner then plans the hand over movements for both robots. For this process, collision free paths to predefined hand-over positions are planned. Depending upon the current position of the robots, the paths are executed in parallel or sequentially to avoid robot collisions.

The above described functionality is encapsulated in two reusable components depicted in Figure 1 and Figure 2. The PlanPath component (Figure 1) tries to compute a complete path including grasping and insertion. Depending on whether it finds a complete path or just a grasping path or no path, the corresponding event output (PATH_FOUND, PATH_HANDOVER_FOUND and NO_PATH_FOUND) is triggered (see Figure 1). In addition, it supports calculating an insertion path from a hand over position.

**
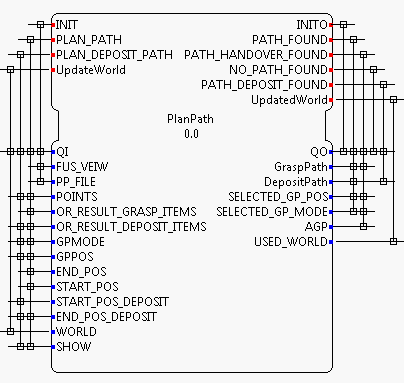
**

***Figure 1:*** *Manipulation Planner Component – Path Plan*

The PathPlanningPointToPoint component (Figure 2) is used to plan paths from any starting position (in most cases the current position of the robot) to any end position. For the benchmark system, this component is used to plan the path from a starting position to the handover position.


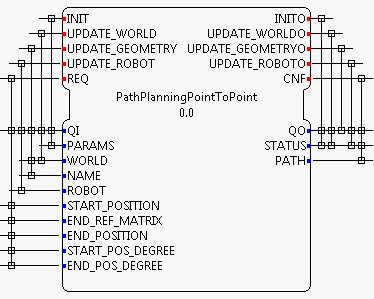


***Figure 2:*** *Manipulation Planner – Plan Path Point to Point*

**C. Robots and Gripper Control Module**: To control the industrial robots of the types Stäubli TX90 and RX130, the software library *LibRoboMove* (which is a part of Profactor’s Robotics and Vision library collection) is used. A ModbusTCP connection is established to send commands and receive status information from the Stäubli robot controllers (7). Thus, sending a set of joint configurations in sequence, resulting in a movement path, is possible. The two-jaw grippers of type SCHUNK PowerCube (8), used for grasping operations, are controlled via their CAN interface, using the manufacturer provided PowerCube API. By using force (current) based gripper movements, an active feedback from the grippers can be interpreted, which is used to detect if a grasp operation was successful.

**Software Architecture of the Benchmark System**

The system architecture of the benchmark system consists of functional modules described above which are coordinated through an application logic, to fulfill the defined tasks. Figure 3 depicts a scheme of the software architecture used for the benchmark system. Arrows depict communication relations between the functional modules and the overall application logic. Every functional module is accessed through an IEC 61499 compliant function block (FB). The role of the application logic is to perform intermediate data processing steps, and also data and event distribution among the modules, thus coordinating the functional modules. The communication relations between functional modules and the application logic are summarized below.

- A point cloud of the scene is acquired by fetching frames from the sensor. The received scene data is pre-processed for noise reduction.
- Based on the pre-processed sensor data, the 3D vision system tries to find the transformations matrices for the objects of interest as described in section A above.
- The resulting object transformation matrices are used to plan collision free paths to grasp fuses and to insert them into the fuse box holes. Moreover, collision free handover paths are planned to enable interaction of two robots.
- For the path execution itself, the generated paths are split into segments. This is necessary to operate the two-jaw robot grippers at the right time (e.g. between reaching a fuse and moving a fuse). The resulting path segments are forwarded to the robot controller (RoboMove library) for execution. The gripper signals are sent to the PowerCube gripper module.

***Figure 3:*** *Software architecture of the benchmark system*

The application logic in Figure 3 is required to coordinate the functional modules and additionally includes functionalities as follows:

- Routing data and events to the functional modules according to the required execution sequence of functions.
- Preparation of collision models for the path planning system based on current robot positions.
- Sensor data pre- and post-processing based on given library functions for point cloud manipulation.
- Application logic to react on events, like user intervention, insertion/grasp failures.

# Learning of Reward Function

Reward structure can also be learnt by the system through experience. The steps in the process go as follows:

1. Initialize the reward values $R_{i}$ of all neurons of a GNG to 1.
2. Create a scenario with two target objects; one of them placed somewhere in the middle of shared workspace, and the other one somewhere in the middle of rest of the peripersonal space of the robot.
3. Find the two most active neurons in the GNG best representing the corresponding object locations. Let these neurons be neuron $j$ and$k$ respectively.
4. Let the robot randomly choose one of the two neurons, since both fetch same reward.
5. If the robot chooses neuron $j$ (neuron representing the object in shared area), penalize the neuron. Spread the penalty throughout the network as a decreasing function of distance of a neuron $i$ from the penalized neuron $j$and stop.

If $U$ is the penalization, then the reward value $R_{i}$ of a neuron $i$ in the network is given by

$$R_{i}=R_{i}-U\frac{1}{\surd2\pi\sigma_{s}}e^{{-\left( s_{i}-s_{j} \right)}^{2}/2\sigma_{s}^{2}}$$

If the robot chooses neuron $k$, repeat steps 2-5.

This process will result in a reward function like the one shown in Figure 4B for TX robot. In fact, it is possible that a network learns the reward field in a single run of the above described process if the robot chooses neuron *j*. A similar reward function can be learnt for RX using the same process.


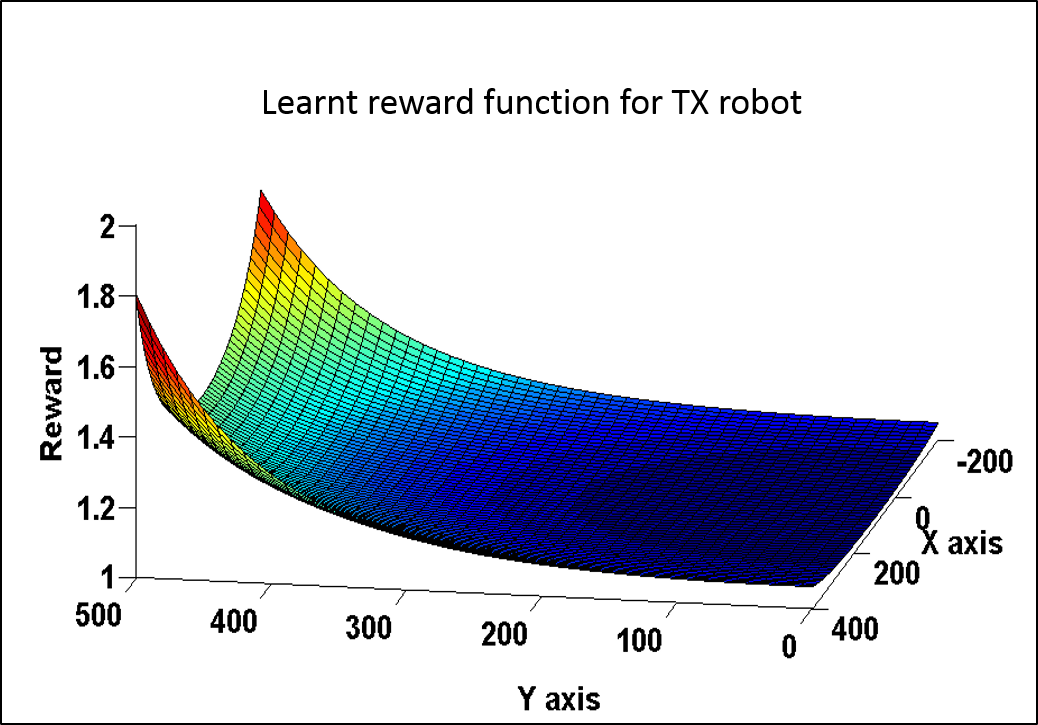


**Figure 4** shows an example of the structure of a reward function attained through learning using the process described in the text.

# Open Source code and documentation

An open source software implementation (in C++) of the model and a user manual is available under GPL license in the GitHub repository at <https://github.com/Vishuu-IIT/DarwinCognitiveArchitecture>. The source code module (named *PMPRX_Neural*) is inside the subdirectory of *DarwinCognitiveArchitecture/tree/master/src/control folder*, while *DarwinCognitiveArchitecture/tree/master/app/RXneural* contains all the configuration files for loading the neural networks and codes for data-generation and training of the neural networks. The implementation uses YARP platform (<http://wiki.icub.org/yarpdoc/>) for communication of messages. The user manual can be found inside *DarwinCognitiveArchitecture/tree/master/documentation* folder. The manual gives a detailed description on how to run the modules and how to use the available code for training new neural networks. In addition, videos of the robots performing assembly tasks are provided on the YouTube channel <https://www.youtube.com/playlist?list=PLIfoHEM1gr26bRcDumdp1hLy42qOsd_K2> as supplementary material.

# References

1. DARWIN Consortium. Deliverable D9.5: Industrial assembly demonstrator and final evaluation. Vienna, Austria; 2015.

2. CANDELOR: Understand 3D [Internet]. 2017 [cited 2017 Aug 7]. Available from: http://candelor.com/

3. Papazov C, Burschka D. An efficient RANSAC for 3D object recognition in noisy and occluded scenes. In: Lecture Notes in Computer Science (including subseries Lecture Notes in Artificial Intelligence and Lecture Notes in Bioinformatics). 2011. p. 135–48.

4. Muja M, Rusu RB, Bradski G, Lowe DG. REIN - A fast, robust, scalable recognition infrastructure. In: Proceedings - IEEE International Conference on Robotics and Automation. 2011. p. 2939–46.

5. Jr JK, Lavalle SM. RRT-Connect An Efficient Approach to Single Query Path Planning. Robot Autom 2000. 2000;54(2).

6. Capco J, Rooker M, Pichler A. RRT planner for the binpicking problem. In: 9th International Workshop on Robot Motion and Control, RoMoCo 2013 - Workshop Proceedings. 2013. p. 154–60.

7. Stäubli Robotics [Internet]. 2017 [cited 2017 Aug 7]. Available from: http://www.staubli.com/en/robotics/

8. SCHUNK [Internet]. SCHUNK: Servo Electric 2-Finger-Parallel Gripper Type PG 70 Assembly and Operating Manual. 2017 [cited 2017 Aug 7]. Available from: https://www.roscomponents.com/en/index.php?controller=attachment&id_attachment=180
